# Paroxysmal Hæmoglobinuria

**Published:** 1910-08-27

**Authors:** 


### Medicine.

### PAROXYSMAL HÆMOGLOBINURIA.

It is possible for a patient suffering from paroxysmal hæmoglobinuria to pass urine having absolutely different characteristics within a single period of twenty-four hours. For instance, one specimen may be quite normal—clear and pale straw-coloured, having a specific gravity of 1015, and containing no albumen, no blood, and a very scanty deposit, consisting merely of mucus. The second specimen may be dark porter-coloured, acid, of specific gravity 1015; it may contain a large quantity of coagulable proteid, and much colouring matter derived from the blood; it may deposit a very abundant dark-brown sediment which on microscopical examination will be found to be composed mainly of brown-coloured granular débris which is not dissolved on heating, a considerable number of renal tube-casts, some of which are hyaline, whilst others are filled with the amorphous brown sediment of which the urinary deposit is chiefly composed, amorphous urates, and a few crystals of oxalate of lime, and also a few, but very few, red blood corpuscles. The third specimen may be pale sherry-coloured, of specific gravity 1015, but may give a very faint blood reaction with the guaiacum and ozonic ether test, though in other respects it may seem normal.

### Dr. Bramwell's Case.

Many cases of the kind have been described; most of the patients have been men, and many of them have had a previous history of syphilis. Dr. Byrom Bramwell, in his "Clinical Studies," records the case of a man thirty-six years of age, a cellarman by occupation, married, who had been under observation for a considerable time, having first complained of the passing of dark-coloured urine which dated from two and a half years back. Upon inquiring into the history it was found that at the age of twenty-six he had had an attack of rheumatic fever, but had otherwise had good health until the occurrence of the present disease. He stated that he had not had syphilis, never lived abroad, and had not had malaria.

His first attack of paroxysmal hæmoglobinuria occurred two and a half years before he first came under observation, and he attributed it to a severe wetting and exposure to cold. During the following winter he had six attacks, and thereafter was free from the condition until January two years later, when another attack occurred after exposure to cold, and during January and February the patient had four attacks, which again cleared up to recur again in the following January.

All the above attacks he attributed to exposure to cold, each commencing with a chilly and weak feeling. There had been no pain in the back and no jaundice, but during some of the attacks he had suffered from headache and febrile disturbance. During the paroxysms he had noticed that there had sometimes been increased frequency of micturition and a feeling of discomfort and irritation in the region of the bladder and along the course of the urethra. Repeated attacks caused him to lose his colour and feel extremely weak, while in the intervals he stated that he felt quite well, and the urine was normal in appearance.

The first specimen of urine passed while in hospital was quite normal, but after remaining out of doors for a short time on a very cold day the patient suddenly felt very shivery, and the next specimen of urine passed was porter-coloured. He was then put to bed and remained there until the following morning, when a further examination of the urine showed the latter to contain a small quantity of blood pigment, but in other respects to be normal.

### Incidence of the Condition.

Paroxysmal hæmoglobinuria is a rare disease, and in some cases presents extremely interesting characteristics; it occurs chiefly in adults, and is almost entirely confined to males; one case, however, has been recorded in which the condition occurred in a girl aged fifteen.

The condition is characterised by the passage of urine containing an abundance of blood pigment. The copious deposit that was present in the second specimen mentioned above contained very few red blood corpuscles, but was chiefly composed of granular débris and dark urates, and a considerable number of tube-casts in which brownish-coloured pigmented granules were noticed. The condition is easily distinguished from hæmaturia, in that in the latter the red colour of the urine is due to the presence of blood corpuscles in the urine.

### A Symptom not a Disease.

Hæmatinuria, or hæmoglobinuria, as it is also termed, is like hæmaturia, a symptom, not a disease. The condition is due to rapid disintegration of red blood corpuscles in the general circulation and the passage through the kidney of altered blood pigment; by the use of the spectroscope it will be seen that the pigment in the urine in cases of hæmoglobinuria is usually either oxyhæmoglobin or methæmoglobin. Microscopical examination of the urine in many cases fails to show any red blood corpuscles, but in some cases there are a few which, if present, are altogether insufficient to account for the coloration of the urine and for the amount of blood pigment which is present.

The most essential features in a case of hæmoglobinuria are the presence in the urine of blood pigment and of globulin; the urine is usually acid, the specific gravity varies considerably, in some cases being low, and in others high; the deposit is generally very copious and of a reddish-brown or dark-brown colour, and consists of brown granular débris with numerous tube-casts often containing brown amorphous granules; crystals of hæmatoidin have been seen in exceptional cases, whilst brown-coloured urates, and crystals of oxalate of lime and of uric acid may also be present in the deposit.

When the urine is heated a copious precipitate often forms, and it occasionally tends to collect at the top of the specimen, and thus differs from the coagulum of serum albumen, which usually falls to the bottom.

Hæmoglobinuria is, as has been stated above, due to the excretion by the kidneys of pigment derived from the red blood corpuscles. This pigment is liberated in the general circulation as the result of destruction of the red blood cells, or possibly in some cases the escape of the blood pigment from the red cells without actual destruction of the corpuscles; it is generally agreed that the essential feature of hæmoglobinuria is the rapid destruction of red blood corpuscles in the general circulation, and this may be due to a number of different conditions, and may occur in connection with a variety of diseases. The transfusion into the blood of one animal of the blood of an animal of a different genus is followed by rapid disintegration of the transfused blood and the production of hæmoglobinuria. If a sufficient amount of lamb's blood is transfused into a man, hæmoglobinuria results, and the injection into the blood of a great number of chemical substances, such as chlorate of potash, glycerine, naphthol, and so forth, will also produce hæmoglobinuria. Quinine has sometimes been known to produce hæmoglobinuria in certain cases, and also to cause blackwater fever, in which the urine is dark-coloured as the result of hæmoglobinuria.

### Ætiology.

Exposure to extreme heat and extreme cold has been known to produce hæmoglobinuria in persons who are not subject to paroxysmal hæmoglobinuria.

Hæmoglobinuria may occasionally occur in the course of certain specific fevers, such as malaria, scarlet fever, typhoid fever, etc. It is occasionally met with in an epidemic form in young infants, and in these cases is usually associated with jaundice and cyanosis. It has also been noticed in connection with Raynaud's disease, and in this it is much more common in females than it is in males, whilst, as has been mentioned, this is generally the reverse in paroxysmal hæmoglobinuria. In some cases of Raynaud's disease, and also in many of hæmoglobinuria, there is a history of syphilis, and it would be interesting to know whether hæmoglobinuria occurs, and only occurs, in those cases of Raynaud's disease with a history of syphilis.

The onset of the most prominent symptoms in the rare disease, paroxysmal hæmoglobinuria, occurs as the result of exposure to cold, and it is chiefly due to this fact that the attacks mainly occur in the winter and cold weather; it is very exceptional for a case to be met with in the summer, and the condition has therefore been termed " winter " hæmoglobinuria.

The exact pathology of paroxysmal hæmoglobinuria is still obscure, but, as has already been mentioned, it is far more common in men than it is in women, and is generally met with between the ages of fifteen and fifty, but cases have been recorded where the condition has developed in young children and elderly people. Patients suffering from paroxysmal hæmoglobinuria are usually quite well between the attacks, and are not the subjects of any structural change or visceral lesion, but any exposure to cold, such as putting the feet or hands into cold water, is in some cases quite sufficient to cause an attack, and the patient may immediately pass large quantities of blood pigment in the urine.

Some observers regard this rapid destruction of red blood corpuscles in the general circulation, which immediately follows exposure to cold in persons who are subject to the disease, as due to vasomotor changes, the contraction of the blood vessels, vascular spasm, and the accumulation in the blood of an excess of carbon dioxide. It has also been suggested that in cases of hæmoglobinuria the resisting power of the red blood corpuscles is lowered in consequence of some derangement or disease of the blood-forming organs; and if this be so it would be necessary to make an examination of the condition of the bone-marrow in cases of paroxysmal hæmoglobinuria. The condition is rarely fatal, and therefore post-mortem investigations very seldom occur.

The phenomenon of paroxysmal hæmoglobinuria seems to suggest that the exposure to cold acting reflexly through the nervous system so disturbs the metabolism of the body as to produce within the latter some toxin which leads to rapid destruction of the red blood-cells. This would, of course, be much more likely to occur if the resisting power of the red cells were lowered either in consequence of disease of the blood-forming organs, such as the bone-marrow, or some idiosyncrasy on the part of the patient.

The production of a toxin within the body, the result of nervous derangement of the metabolism, possibly of the function of the liver, due to the exposure to cold, seems a much more likely theory than the vascular-spasm and carbon dioxide theory above mentioned.

There is the possibility that the paroxysms of hæmoglobinuria are not altogether due to destruction of the red corpuscles, but to the escape of hæmoglobin from the corpuscles, without the actual disintegration of the red cells, but this point has not been established, and requires further investigation.

### Treatment.

In treating cases of paroxysmal hæmoglobinuria it is most important to keep the general health of the patient in the best possible state, and to see that he is well and warmly clothed. Should the patient's occupation necessitate exposure to cold he would be wise to undertake some indoor work, and, if possible, to live in an equable and warm atmosphere, and patients who could afford it should spend the cold months of the year at the South Coast or abroad.

The patient should be placed in bed immediately an attack occurs, and should remain there for some time; should the patient become anaemic after an attack or series of attacks, iron or a mixture of iron and arsenic should be given, and after convalescence arsenic should be continued with the object of increasing the resisting power of the red blood corpuscles. The application of the *x*-rays to the long bones and spleen may also be beneficial in these cases, and the administration during the intervals between the attacks of quinine, nux vomica, and strychnine has also been found useful.

